# Quantitative Structure-Activity Relationships Predicting the Antioxidant Potency of 17β-Estradiol-Related Polycyclic Phenols to Inhibit Lipid Peroxidation

**DOI:** 10.3390/ijms14011443

**Published:** 2013-01-11

**Authors:** Laszlo Prokai, Nilka M. Rivera-Portalatin, Katalin Prokai-Tatrai

**Affiliations:** 1 Department of Molecular Biology and Immunology, University of North Texas Health Science Center, Fort Worth, TX 76107, USA; E-Mail: nilka.rivera1@upr.edu; 2 Department of Pharmaceutical Sciences, UNT System College of Pharmacy, University of North Texas Health Science Center, Fort Worth, TX 76107, USA; E-Mail: katalin.prokai@unthsc.edu

**Keywords:** 17β-estradiol, estrogens, lipid peroxidation, phenolic antioxidant, oxidative stress, QSAR, TBARS

## Abstract

The antioxidant potency of 17β-estradiol and related polycyclic phenols has been well established. This property is an important component of the complex events by which these types of agents are capable to protect neurons against the detrimental consequences of oxidative stress. In order to relate their molecular structure and properties with their capacity to inhibit lipid peroxidation, a marker of oxidative stress, quantitative structure-activity relationship (QSAR) studies were conducted. The inhibition of Fe^3+^-induced lipid peroxidation in rat brain homogenate, measured through an assay detecting thiobarbituric acid reactive substances for about seventy compounds were correlated with various molecular descriptors. We found that lipophilicity (modeled by the logarithm of the *n*-octanol/water partition coefficient, logP) was the property that influenced most profoundly the potency of these compounds to inhibit lipid peroxidation in the biological medium studied. Additionally, the important contribution of the bond dissociation enthalpy of the phenolic O–H group, a shape index, the solvent-accessible surface area and the energy required to remove an electron from the highest occupied molecular orbital were also confirmed. Several QSAR equations were validated as potentially useful exploratory tools for identifying or designing novel phenolic antioxidants incorporating the structural backbone of 17β-estradiol to assist therapy development against oxidative stress-associated neurodegeneration.

## 1. Introduction

Oxidative stress (OS) has been implicated as a key factor to the initiation and/or progression of numerous pathological processes and premature aging [1]. OS-initiated lipid peroxidation (LPO) has been extensively studied as a potential biomarker for this detrimental process, since lipids are particularly sensitive to OS [2,3]. The high unsaturated lipid content of the brain, coupled with high oxygen utilization, elevated redox-active metal ions concentration and relatively poor antioxidant defense, makes this organ quite vulnerable to this process and the associated pathologies [4]. One of the most promising approaches to combat OS and, thus, prevent the resultant LPO is the use of antioxidants that can either “catch” free radicals or chelate excessive redox metals that can provoke OS via the formation of reactive oxygen species (ROS) [5].

**Figure 1:**
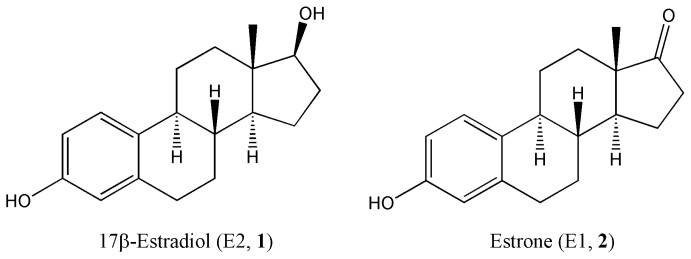
Chemical structure of the 17β-estradiol (E2) and estrone (E1).

Estrogens are unique among steroids in terms of having the phenolic A-ring that allows for eliciting antioxidant activity similar to that of simple phenolic antioxidants, such as vitamin E or butylated hydroxytoluene [6,7]. It has become evident that the ability of 17β-estradiol (E2, **1**), the main human estrogen (Figure 1), to act as a direct free radical scavenging antioxidant is an important element in the cascade of neuroprotective events by which E2 provides protection against OS both *in vitro* [7–9] and *in vivo* [10–12], although the latter is not without controversy [13]. Nevertheless, it has been shown that E2 and related compounds are able to directly scavenge free radicals and, thereby, alleviate OS independently of nuclear estrogen receptor (ER) activation [14,15]. Only the free phenolic OH of the A-ring is the prerequisite for this activity. This seminal finding was first recognized by Behl *et al.* [16]. An important new antioxidant cycle for estrogens to shield against free radicals was recently discovered showing that estrogens can actually be regenerated after sweeping up free radicals [10,17]. Additional proposed mechanisms include the synergistic interaction between estrogens and glutathione [18], as well as chelating redox metals, particularly by estrogens having an unsaturated B-ring [19]. It should also be noted that the indirect antioxidant actions of these biomolecules have also been confirmed; most prominently via the upregulation of the endogenous antioxidant defense system [20].

There are several caveats associated with the use of E2 for antioxidant therapy targeting the brain; for example, E2 lacks adequate oral bioavailability [21] and its administration also produces unwanted hormonal and other potentially detrimental side-effects in the periphery [22,23]. In the search for new E2-derived agents that preserve E2’s strong antioxidant property [24] without peripheral liability, studies with limited scopes have been conducted with E2 derivatives exhibiting diminished or no ER binding affinity [25]. It has been known that manipulation of ER-binding can be done by introducing substituents to and/or performing isomerization on the E2 structure [26,27]. In particular, the fundamental work of Miller *et al*. concluded that the ER-binding affinity (*i.e.*, estrogenic activity) and antioxidant efficacy can be largely separated with the presence of bulky substituents on the phenolic A-ring of E2 [27]. Quantitative structure–activity relationship (QSAR) studies connecting the estrogen structure and ER-binding ability necessary to trigger the well-documented genomic actions of estrogens have been reported, most particularly to identify estrogenic endocrine disruptors [28–30].

*In vitro* studies to establish the structural requirements for antioxidant potency have used small libraries of compounds [15,31,32]. In order to facilitate drug discovery-driven efforts to search and identify potentially useful antioxidants using E2 and estrone (E1, **2**) as templates (Figure 1), we aimed at performing QSAR studies using an extended library of E2 and E1 derivatives or analogues (**3**–**60**), as well as related polycyclic phenols (**61**–**70**). The QSAR modeling was based on the construction of predictive models for the inhibition of Fe^3+^-induced LPO in rat brain homogenate by these compounds [33], assessed through the detection of thiobarbituric acid reactive substances (TBARS), as an experimental measure for antioxidant potency. The TBARS assay is a widely adopted and sensitive method for measuring the extent of LPO [34]. The oxidation of unsaturated fatty acids leads to the formation of malondialdehyde (MDA) as a breakdown product [35]. The reaction of MDA with thiobarbituric acid (TBA) produces a pink chromogen when heated at low pH with a typical maximum absorbance at 532 nm [34]. The MDA-TBA complex measured by the TBARS assay is considered a gauge for lipid peroxide (LOOH) formation [36]. Based on this established experimental model, the goal of the present work was to find QSARs that could approximate through a theoretical approach whether a particular estrogen derivative or related phenolic compound would be a more or less potent antioxidant against LPO than E2 (**1**). These models then may serve as exploratory tools for identifying and designing novel phenolic antioxidants via utilizing the structural backbone of the parent steroid.

## 2. Results and Discussion

### 2.1. Construction of QSAR Models

The experimental antioxidant potencies, expressed as IC_50_ values for the inhibition of LPO measured via the TBARS assay in ovariectomized (OVX) rat brain homogenate against Fe^3+^-induced LPO [25,37,38], of the selected compounds (**1**–**70**) are given in the Supplementary Information (Table S1, which is a spreadsheet in Microsoft Excel format also displaying the chemical structures). Specifically, IC_50_ of a compound represents the concentration that inhibits 50% of LPO; thus, a smaller number represents a higher potency in this regard. In comparison with alternative chemometric and cheminformatics tools, the advantage of a descriptor-based approach for the development of predictive QSAR models focusing on LPO inhibitory activity has been shown recently [39]. Therefore, we applied the latter strategy to pursue our computational study reported here. The negative logarithm of the IC_50_ value (in molar concentration, M) was chosen as the dependent variable, and various descriptors of the test compounds available through the Project Leader module of the CAChe software were considered as independent variables for the creation of QSAR models:

(1) $$-\text{log}({\text{IC}}_{50}-\text{TBARS})=13.6\;(\pm 3.4)\;-0.179\;(\pm 0.042)\cdot \text{BDE }(r=0.46,F=18.11,p<0.001)$$

(2) $$-\text{log}({\text{IC}}_{50}-\text{TBARS})=-2.69\;(\pm 0.36)+0.118\;(\pm 0.021)\cdot {\text{SI}}_{\kappa 1}\;(r=0.55,F=29.85)$$

(3) $$-\text{log}({\text{IC}}_{50}-\text{TBARS})=-3.03\;(\pm 0.41)+0.00728\;(\pm 0.0031)\cdot \text{SA }(r=0.56,F=30.97)$$

(4) $$-\text{log}({\text{IC}}_{50}-\text{TBARS})=-2.27\;(\pm 0.27)+0.295\;(\pm 0.052)\cdot \text{logP }(r=0.57,F=32.42)$$

(5) $$-\text{log}({\text{IC}}_{50}-\text{TBARS})=6.48\;(\pm 3.42)+0.237\;(\pm 0.0547)\cdot \text{logP}-0.105\;(\pm 0.041)\cdot \text{BDE}\;(r=0.58,F=20.83)$$

(6) $$-\text{log}({\text{IC}}_{50}-\text{TBARS})=6.23\;(\pm 2.63)+0.288\;(\pm 0.048)\cdot \text{logP}+0.988\;(\pm 0.304)\cdot \text{HOMO }(r=0.64,F=23.78)$$

(7) $$-\text{log}({\text{IC}}_{50}-\text{TBARS})=2.56\;(\pm 0.36)+0.186\;(\pm 0.102)\cdot \text{logP}+0.0522\;(\pm 0.0421)\cdot {\text{SI}}_{\kappa 1}\;(r=0.58,F=17.10)$$

(8) $$-\text{log}({\text{IC}}_{50}-\text{TBARS})=18.5\;(\pm 4.3)+0.209\;(\pm 0.050)\;\text{logP}-0.130\;(\pm 0.037)\cdot \text{BDE}+1.16\;(\pm 0.29)\cdot \text{HOMO }(r=0.71,F=22.60)$$

The best statistical models are shown in Equations 1–8. We believe that the somewhat modest correlations were due to the combination of limited structural diversity in the training set and confines of the *in vitro* experimental procedure relying on an actual, heterogeneous biological medium [33] rather than a well-defined chemical model for LPO [40]. Nevertheless, all of them satisfied the requirement for statistical significance with *p* < 0.001 from analysis of variance (ANOVA). The values of phenolic O–H’s bond dissociation enthalpy (BDE, kcal/mol), a shape index (κ-type, first order, SI_κ1_), the solvent-accessible surface area (SA, Å^2^), lipophilicity (expressed as the logarithm of the *n*-octanol/water partition coefficient; logP), and the eigenvalues of a frontier orbital (HOMO, eV) were the descriptors present in the QSAR models obtained, and these descriptors were also included in Table S1.

The first four equations represent models created from the use of only a single molecular descriptor. Equation 4 provided the largest *F*-value (the ratio of the model’s explained variance to its unexplained variance, considering *F* of 15 as threshold value for model selection). This indicated that logP (*i.e.*, a descriptor related to lipophilicity) had the best predictive value among parameters found to give the best one-descriptor Equations (1–4; BDE, SI_κ1_, SA and logP), confirming thereby the previously established significance of lipophilicity regarding LPO inhibition [40]. In addition, logP was a steady descriptor included, when equations of acceptable statistical significance were searched using two or more independent variables, while other variables in these QSAR models were either BDE (Equation 5), HOMO (Equation 6), SI_κ1_ (Equation 7), or BDE and HOMO (Equation 8), respectively. Altogether, inclusion of descriptors other than logP in Equations 5–8 decreased the F-values but, with the exception of including SI_κ1_ (Equation 7), improved correlation (*i.e.*, increased the r value).

**Figure 2:**
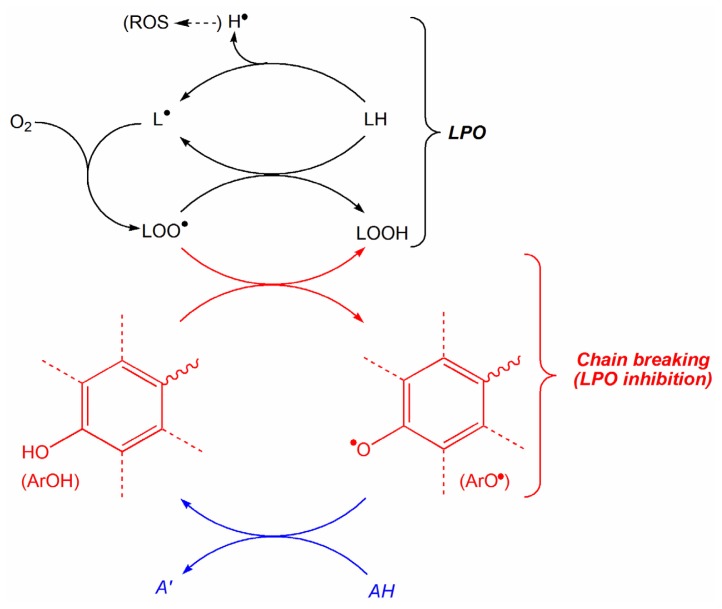
Schematic illustration of lipid peroxidation (LPO) inhibition by phenolic antioxidants.

The extended spin distribution in the phenoxyl radical (ArO^•^) derived from the parent phenolic antioxidant (ArOH; e.g., E2) after it donates its H from the phenolic OH to a free radical to terminate the propagation of a radical reaction has been suggested to be an important contributor for the radical scavenging activity [41,42]. A smaller value of this parameter projects a more stable ArO^•^ and, consequently, better antioxidant potency [40]. Nevertheless, correlation of BDE with extended spin distribution, as well as with the enthalpy of single-electron transfer and the ionization potential (IP) were also noted [40]. Therefore, the BDE was considered for the construction of QSAR models in this context. The BDE increases with the increasing electron withdrawal by the substituents surrounding ArOH; in other words, O–H bond is weakened by increasing the electron density and strengthened by decreasing the electron density within the bond [43]. Accordingly, electron donating group(s) on the A-ring of an estrogen should positively impact the antioxidant potency compared to that of the unsubstituted E2 (**1**). Concurring, Equations 1, 5 and 8 correctly predict that the inhibition of LPO decreases with increasing BDE. This tendency is expected, because compounds that can easily donate the hydrogen of the phenolic OH to break the cascade of radical-mediated reactions are those with low BDE. This process is schematically shown in Figure 2 [44]; where LH represents a lipid molecule, LOO^•^ is the product of a very fast O_2_-addition to the chain initiator [45] formed by the reaction LH → L^•^ upon the attack by ROS, and LOOH is lipid hydroperoxide. The chain-breaking reaction ArOH + LOO^•^ → ArO^•^ + LOOH prevents LPO cycle propagated by an H-atom exchange reaction (LOO^•^ + LH → LOOH + L^•^) that regenerates the chain initiator. The pathway drawn in red represent the actual chain-breaking H-atom transfer, while the blue portion of Figure 2 implicates the conversion of the phenoxyl radical (ArO^•^) back to the phenolic compound (ArOH) by an endogenous reductant AH such as ascorbate, which is converted to its oxidized form A’ in the process [44].

The HOMO energy (the energy required to remove an electron from the highest occupied molecular orbital) has also been connected to the ability of a phenolic compound to donate electrons to free radicals. According to Koopman’s theorem and the molecular orbital theory [40], it determines the IP. Therefore, the involvement of the HOMO energy as descriptor refining two different QSAR models (Equations 6 and 8, respectively) was not unexpected, although it did not qualify alone to be among the one-parameter equations giving statistically acceptable correlation. Nevertheless, it was noticeable that correlation increased when this descriptor was also used in the two-parameter equations in addition to logP and BDE, respectively. It is noteworthy that the best three-parameter equation also included HOMO (Equation 8) and, provided a larger r-value than BDE and logP without this descriptor (Equation 5). The influence of a topological index related to the shape of a molecule was also revealed. The SI_κ1_ quantifies the number of cycles in the compound [46,47]. Equations 2 and 7 predict that a large SI_κ1_ value improves the antioxidant potency of an E2-related polycyclic phenol. SA also gave a good correlation and had apparent descriptive value considering the TBARS assay as a measure of LPO inhibition by these compounds. These descriptors were calculated at an optimized geometry in water using the conductor-like screening model (COSMO) for solvation [48]. Equation 3 suggests that a higher antioxidant activity could be obtained with molecules having a higher SA.

### 2.2. Validation of QSAR Models (Equations 1–8)

An important step in the QSAR modeling is to validate the obtained models. An initial validation was carried out for the best models (Equations 1–8) through randomly leaving out 10% of compounds from the training set [49,50] (data not shown). The equations fitted to this reduced training set with the same descriptors yielded slightly different regression coefficients, but the correlation coefficients of the equations remained similar to those obtained with all entities (**1**–**70**) included. In addition, the lack of chance correlations in the reported QSAR models was also ensured by analyzing the equations after randomization of the experimental values.

In a more rigorous validation that was independent from our training set, the obtained QSAR equations were used to predict the relative antioxidant potencies of A-ring substituted estrogens and compared with those obtained by Badeau *et al.* [32]. These authors analyzed a group of estrogens for their antioxidant capacity and reported the potencies of the compounds as more active or less active than E2 (**1**). The IC_50_’s for the TBARS of these compounds were predicted using QSAR Equations 1–8 and compared with the results reported. The validity of the predictions was determined based on the number of false positives (compounds that were less potent than E2 but were predicted to be more potent), false negatives (compounds that are more potent than E2 but were predicted to be less potent) and well-predicted compounds (Table 1). Even though the experimental approach by Badeau *et al.* [32] was not based on the TBARS method but assessed the antioxidant effect on copper-induced oxidation of lipoproteins through the continuous monitoring of conjugated diene formation, our QSAR Equations 1 and 4–8 still correctly predicted the relative antioxidant capacities of A-ring substituted estrogens. Therefore, the strong influence of logP, as well as the contribution of BDE, SI_κ1_ and HOMO was certainly confirmed in a general context of LPO inhibition by these E2-related compounds.

## 3. Experimental Section

### 3.1. Materials and Methods

Triton X-100, EDTA, bovin serum albumin (BSA), copper (II) sulfate, bicinchoninic acid (BCA), iron (III) chloride, trichloroacetic acid, hydrochloric acid and thiobarbituric acid (TBA) were purchased from Sigma-Aldrich (St. Louis, MO, USA). All other chemicals and solvents were purchased from Fisher Scientific Company (Pittsburgh, PA, USA). Brains were obtained from 2 to 6 ovariectomized (OVX) Sprague-Dawley rats (Charles River Laboratories, Wilmington, MA, USA), three weeks after ovariectomy to ensure that endogenous E2 and E1 would be negligible in the animals and, therefore, would not be interfering factors in the *in vitro* studies. All animal procedures were approved by the Animal Care and Use Committee of University of North Texas Health Science Center.

The training data set used to construct the models for the QSAR study was composed of seventy compounds. These compounds were commercially available (Steraloids, Newport, RI or Sigma-Aldrich, St. Louis, MO, USA), or their availability has been specified earlier [25]. They are listed in Table S1, along with their experimentally determined antioxidant potencies against iron(III)-induced LPO in OVX rat brain homogenate expressed as IC_50_ values (in molar concentration, M) relying on the TBARS method [25,37]. Protein concentrations were determined through bicinchoninic acid assay [51,52]. Briefly, 20% *w*/*v* rat brain homogenate [53] (prepared in aqueous TritonX buffer: TritonX-100, 1% *v*/*v*; EDTA 1 mM; NaCl, 0.9% *w*/*v*) was diluted into phosphate-buffered saline, pH 7.4, to afford 1 mg/mL protein concentration. After addition of the test compound from ethanolic stock solution, the mixture was held at room temperature for 30 min. FeCl_3_ was then added from aqueous stock solution to reach 300 μM in its concentration, and the sample was incubated at 37 °C for 15 min. For the measurement of MDA formation, 150 μL of 12.5% (*v*/*v*) trichloroacetic acid in 0.8 N HCl and 300 μL TBA (1%, *w*/*v*) solutions were added and incubated for an additional hour at 37 °C. Then, the sample was centrifuged at 12,000 rpm for 2 min. The relative fluorescent units (RFU) of the supernatant was determined at an excitation and emission wavelengths of 530 and 590 nm respectively in a fluorescence FL600 microplate reader (Biotek, Winooski, VT, USA). The percent of inhibition of LPO from the TBAR assay was calculated as follows:

(9) $$\%\;\text{inhibition}=(1-A/A_{0})\times 100$$

where *A* is the absorbance in the presence of the antioxidant compound at various concentrations and *A*_0_ is the absorbance of the control reaction. Each compound was tested in three independent experiments with five to six different levels of inhibitor concentration in each experiment. Sigmoidal dose–response relationships were presumed. Prism (version 3.0; GraphPad Software, La Jolla, CA, USA, 2005) was used to calculate the IC_50_ values of the compounds.

### 3.2. QSAR

Computations were performed by using CAChe software (version 6.1.1; Fujitsu, Beaverton, OR, USA, 2006). Structures were preoptimized using augmented MM3 parameters followed by full geometry optimization (RMS gradient <0.1 kcal/(Å·mol)) and energy minimization by using the PM5 semi-empirical method with open-shell wave functions (unrestricted Hartree-Fock (UHF) approach).

QSAR equations were obtained through the Project Leader molecular spreadsheet linked to CAChe. In addition to “built-in” structural, spatial, electronic, quantum-chemical and thermodynamic descriptors available through this module, BDE of the phenolic O–H bond [40] was also included for each compound. The latter descriptor was calculated as

(10) $$\text{BDE}={\text{H}}_{\text{r}}+{\text{H}}_{\text{H}}-{\text{H}}_{\text{p}}$$

where H_p_ and H_r_ were the calculated enthalpies of formation for the parent phenolic molecule and for the phenoxyl radical, respectively, while an experimental value (52.08 kcal/mol) was used as the enthalpy of formation for the hydrogen atom (H_H_). The stepwise regression algorithm of the Project Leader module was applied to select appropriate descriptors for building the QSAR models. ANOVA was performed using the Minitab software (version 14; Minitab Inc., State College, PA, USA, 2005) to obtain the *F*-values for the regressions.

## 4. Conclusions

The main finding of our QSAR study reported here is that lipophilicity was the property that influenced most profoundly the potency of estrogen derivatives and related polycyclic phenols to inhibit iron(III)-induced LPO in rat brain homogenate. The contribution of BDE of the phenolic O–H group, a shape index (SI_κ1_) and HOMO was also confirmed by our theoretical approach. Several QSAR Equations 1 and 4–8 were validated as potentially useful exploratory tools for identifying or designing novel phenolic antioxidants incorporating the structural backbone of E2 to inhibit LPO and, thus, to assist the discovery of potential therapeutic interventions that could alleviate oxidative stress-induced neurodegeneration in the brain.

## Figures and Tables

**Table 1:** QSAR model validation using data reported by Badeau *et al.* [32]).

Equation Number	False Positives a	False Negatives b	Correctly Predicted c
1	6	1	21
2	19	1	8
3	17	1	10
4	2	3	23
5	5	1	22
6	6	2	20
7	1	7	20
8	7	3	18

a False positives are compounds that are less potent than E2 but were predicted to be more potent;

b False negatives are those compounds that are more potent than E2 but were predicted to be less potent; and

c Correctly-predicted are those compounds that were predicted correctly as more or less potent than E2 [32]. Leaving out Equations 2 and 3 that were definitely not validated by this strategy, the overall rate of correct predictions was 74%, while false positives and false negatives were 16% and 10%, respectively.

## Supplementary Files

Supplementary File 1 Supplemental Table (XLSX, 367 KB)
